# Automated Analysis of Diffusion‐Weighted Magnetic Resonance Imaging for the Differential Diagnosis of Multiple System Atrophy from Parkinson's Disease

**DOI:** 10.1002/mds.28281

**Published:** 2020-09-16

**Authors:** Florian Krismer, Vincent Beliveau, Klaus Seppi, Christoph Mueller, Georg Goebel, Elke R. Gizewski, Gregor K. Wenning, Werner Poewe, Christoph Scherfler

**Affiliations:** ^1^ Department of Neurology Medical University of Innsbruck Innsbruck Austria; ^2^ Neuroimaging Research Core Facility Medical University of Innsbruck Innsbruck Austria; ^3^ Department of Medical Statistics, Informatics and Health Economics Medical University of Innsbruck Innsbruck Austria; ^4^ Department of Neuroradiology Medical University of Innsbruck Innsbruck Austria

**Keywords:** multiple system atrophy, MRI, diffusion‐weighted imaging, Freesurfer

## Abstract

**Background:**

Manual region‐of‐interest analysis of putaminal and middle cerebellar peduncle diffusivity distinguishes patients with multiple system atrophy (MSA) and Parkinson's disease (PD) with high diagnostic accuracy. However, a recent meta‐analysis found substantial between‐study heterogeneity of diagnostic accuracy due to the lack of harmonized imaging protocols and standardized analyses pipelines.

**Objective:**

Evaluation of diagnostic accuracy of observer‐independent analysis of microstructural integrity as measured by diffusion‐tensor imaging in patients with MSA and PD.

**Methods:**

A total of 29 patients with MSA and 19 patients with PD (matched for age, gender, and disease duration) with 3 years of follow‐up were investigated with diffusion‐tensor imaging and T1‐weighted magnetic resonance imaging. Automated localization of relevant brain regions was obtained, and mean diffusivity and fractional anisotropy values were averaged within the regions of interest. The classification was performed using a C5.0 hierachical decision tree algorithm.

**Results:**

Mean diffusivity of the middle cerebellar peduncle and cerebellar gray and white matter compartment as well as the putamen were significantly increased in patients with MSA and showed superior effect sizes compared to the volumetric analysis of these regions. A classifier model identified mean diffusivity of the middle cerebellar peduncle and putamen as the most predictive parameters. Cross‐validation of the classification model yields a Cohen's κ and overall diagnostic accuracy of 0.823 and 0.914, respectively.

**Conclusion:**

Analysis of microstructural integrity within the middle cerebellar peduncle and putamen yielded a superior effect size compared to the volumetric measures, resulting in excellent diagnostic accuracy to discriminate patients with MSA from PD in the early to moderate disease stages. © 2020 The Authors. *Movement Disorders* published by Wiley Periodicals LLC on behalf of International Parkinson and Movement Disorder Society

## 1 Introduction

Multiple system atrophy (MSA) is an orphan and progressive neurodegenerative disorder featuring parkinsonian, cerebellar, and autonomic symptoms.^1^ Symptoms of early‐stage MSA frequently mimic Parkinson's disease (PD), hampering an early differential diagnosis.^2^, ^3^, ^4^ Accuracy rates of a clinical diagnosis of PD may range from 65% to 93%.^2^, ^5^, ^6^ However, a reliably early differential diagnosis is critical to patient counseling and for recruitment into interventional trials and other types of academic research.

Numerous magnetic resonance imaging (MRI) studies attempted to improve the diagnostic accuracy for the early differential diagnosis of parkinsonian disorders.^7^ Recently, advances in MRI postprocessing algorithms have provided an opportunity to localize and grade MSA‐specific brain atrophy patterns.^8^, ^9^, ^10^, ^11^, ^12^ However, prior to tissue loss becoming measurable as brain atrophy, the neurodegenerative cascade causes dysfunction at the cellular level, including membrane destruction. Alterations of tissue integrity can be quantified by diffusion‐tensor MRI (DTI).^13^, ^14^ DTI estimates the microstructural integrity and the degree of axonal pathology in brain tissue by quantifying the amount and direction of diffusing water molecules.^13^, ^15^ DTI signal changes in the putamen and the middle cerebellar peduncle (MCP), assessed by manually labeled regions of interest (ROI), were shown to differentiate the Parkinson variant of MSA from PD reliably.^16^, ^17^, ^18^, ^19^ However, a meta‐analysis reported substantial methodological differences, including variability of manual ROI placement, causing a considerable between‐study heterogeneity.^20^

In the present study, we sought to apply established automated subcortical volume segmentation to precisely delineate relevant brain regions and estimate averaged DTI parameters within these ROIs in a uniform fashion by observer‐independent colocalization. We then evaluated the diagnostic accuracy of this multimodal approach for the differential diagnosis of MSA and PD.

## 2 Materials and Methods

### 2.1 Patients

Patients with MSA and PD from the MRI database of the Movement Disorders outpatient clinic at the Medical University of Innsbruck were identified based on the following inclusion criteria: (1) a clinical diagnosis of probable MSA (all patients showed some degree of parkinsonism) or PD at the last visit according to consensus operational criteria,^21^ ^,^ ^22^(2) a clinical follow‐up of ≥36 months, and (3) the availability of high‐quality DTI registrations. Exclusion criteria were white‐matter lesions grades 2 and 3, vascular or space‐occupying lesions within the cerebrum, or motion artifacts on MRI. Presynaptic nigrostriatal dopaminergic dysfunction was confirmed by dopamine transporter single photon emission computed tomography in all patients with MSA. The present cohort represents a subset (those patients who have DTI registrations of sufficient quality) of a previously published cohort.^9^

The clinical evaluation included the Hoehn and Yahr staging scheme, the Unified Parkinson's Disease Rating Scale, and the Mini‐Mental State Examination. All rating scales in patients with PD were performed in the *on* state.

### 2.2 Magnetic Resonance Sequences

MRI measurements were performed on a 3.0 Tesla magnetic resonance scanner (Magnetom Verio, Siemens, Erlangen, Germany) equipped with a 12‐channel head coil. All participants underwent the same MRI protocol, including a coronal 3‐dimensional T1‐weighted magnetization prepared rapid gradient echo and a transversal diffusion‐weighted echo‐planar imaging with diffusion‐sensitizing gradients in 20 directions with a b value of 1000  s/mm^2^ and 1 reference image with b = 0 s/mm^2^.

Segmentation of the subcortical regions and estimation of structure volumes were performed using the FreeSurfer package 6.0 (http://surfer.nmr.mgh.harvard.edu/).^23^, ^24^, ^25^, ^26^ Moreover, a published technique to integrate MCP segmentation into FreeSurfer was applied.^9^ The preprocessing steps were visually inspected to ensure that no misalignment of brain structures had occurred. We then processed native DTI data and performed structural‐to‐diffusion data coregistration using FreeSurfer and FMRIB Software Library tools. This process included (1) eddy current and motion correction, (2) DTI general linear model fit and tensor construction, (3) registration of low b image to same‐subject anatomical T1, and (4) fractional anisotrophy (FA)/mean diffusivity (MD) mapping to Talairach space.

### 2.3 Data Analysis

Data analyses were performed using R 3.6.1 (R Foundation for Statistical Computing, Vienna, Austria). Gaussian distribution was confirmed by visual analysis of Q‐Q plots and the Kolmogorov‐Smirnov test. Group differences of normally distributed data were analyzed by parametric tests and non‐Gaussian distributed variables by nonparametric tests. Distributional differences were determined by the Pearson chi‐square test for independence. Bonferroni correction for multiple testing was applied where applicable. A C5.0 decision tree to classify patients with PD and MSA informed by MD, FA, and volume measurements from subcortical brain regions was developed (http://www.rulequest.com/see5-info.html). Leave‐one‐out cross‐validation was employed to estimate the classification performance of the decision tree.

## 3 Results

### 3.1 Demographics

A total of 28 patients with MSA (19 Parkinson predominant, 9 cerebellar predominant) and 19 patients with PD as well as 25 age‐matched and sex‐matched healthy individuals were included in the present study. There was no significant difference between the study groups regarding gender distribution, age, and disease duration. Motor impairment was significantly greater in patients with MSA than in patients with PD. Detailed information on the participants' demographics is provided in Table 1.

**TABLE 1:** Demographics and basic clinical information

Variables	HC, N = 25	PD, N = 19	MSA, N = 28	Total, N = 72	*P* Value
Sex, n (%)					0.957^a^
Female	11 (44.0)	8 (42.1)	13 (46.4)	32 (44.4)	
Male	14 (56.0)	11 (57.9)	15 (53.6)	40 (55.6)	
Age at MRI scan					0.048^b^
Mean (SD)	60.0 (5.9)	64.9 (5.8)	63.4 (7.8)	62.6 (6.9)	
Disease duration at MRI					0.194^b^
Mean (SD)	NA	3.1 (1.9)	2.4 (1.7)	2.7 (1.8)	
Predominant motor presentation, n (%)					
Parkinson predominant	NA	NA	19 (67.9)	NA	
Cerebellar predominant	NA	NA	9 (32.1)	NA	
Hoehn and Yahr staging					<0.001^c^
Mean (SD)	NA	2.39 (0.54)	3.30 (0.69)	2.94 (0.77)	
Range	NA	1.50–3.00	2.00–4.00	1.50–4.00	
MMSE					0.124^b^
Mean (SD)	NA	28.7 (1.5)	27.8 (2.2)	28.2 (2.0)	
UPDRS III					<0.001^b^
Mean (SD)	NA	24.6 (6.5)	40.2 (12.9)	33.9 (13.2)	
UPDRS sum score					<0.001^b^
Mean (SD)	NA	37.1 (9.9)	64.2 (19.1)	53.3 (20.8)	

^a^ Pearson chi‐squared test.

^b^ Linear model analysis of variance.

^c^ Kruskal‐Wallis rank‐sum test.

Abbreviations: HC, healthy controls; PD, Parkinson's disease; MSA, multiple system atrophy; MRI, magnetic resonance imaging; SD, standard deviation; MMSE, Mini‐Mental State Examination; UPDRS III, Unified Parkinson's Disease Rating Scale Part III.

### 3.2 Group Analysis of DTI Measures

Group differences of key brain regions and corresponding effect sizes are provided in Table 2. Significantly increased MD and significantly decreased FA values were observed in numerous subcortical brain regions of the MSA group compared with the PD group and healthy controls. There were no group differences between patients with PD and healthy controls. When comparing patients with MSA and PD, the effect sizes were largest within the MCP, the cerebellar cortex, the cerebellar white matter, and the putamen.

**TABLE 2:** Mean diffusivity and fractional anisotropy values of relevant brain regions

DTI measure	HC, N = 25, Mean (SD)	PD, N = 19, Mean (SD)	MSA, N = 28, Mean (SD)	Total, N = 72, Mean (SD)	*P* Value	Effect Size
Mean diffusivity						
Cerebellum, white matter	0.719 (0.023)	0.715 (0.026)	0.862 (0.152)	0.773 (0.119)	<0.001	1.24
Cerebellar cortex	0.945 (0.048)	0.937 (0.040)	1.159 (0.192)	1.026 (0.163)	<0.001	1.47
Putamen	0.747 (0.035)	0.772 (0.034)	1.000 (0.222)	0.852 (0.184)	<0.001	1.32
Middle cerebellar peduncle	0.752 (0.033)	0.744 (0.020)	0.886 (0.124)	0.802 (0.104)	<0.001	1.47
Superior cerebellar peduncle	1.028 (0.179)	1.063 (0.183)	1.461 (0.460)	1.206 (0.377)	<0.001	0.68
Thalamus	0.866 (0.044)	0.892 (0.038)	0.914 (0.059)	0.892 (0.053)	0.003	0.43
Caudate nucleus	0.977 (0.103)	1.005 (0.144)	1.013 (0.153)	0.998 (0.134)	0.611	0.06
Pallidum	0.770 (0.109)	0.774 (0.096)	0.800 (0.106)	0.783 (0.104)	0.534	0.25
Midbrain	0.915 (0.058)	0.914 (0.061)	0.975 (0.079)	0.938 (0.073)	0.002	0.84
Pons	0.850 (0.064)	0.836 (0.050)	0.951 (0.136)	0.886 (0.109)	<0.001	1.05
Fractional anisotropy						
Cerebellum, white matter	0.396 (0.033)	0.389 (0.027)	0.327 (0.054)	0.367 (0.052)	<0.001	1.36
Cerebellar cortex	0.178 (0.014)	0.182 (0.016)	0.155 (0.028)	0.170 (0.024)	0.001	1.10
Putamen	0.248 (0.030)	0.248 (0.026)	0.260 (0.033)	0.253 (0.030)	0.319	−0.37
Middle cerebellar peduncle	0.477 (0.027)	0.483 (0.027)	0.424 (0.048)	0.458 (0.045)	<0.001	1.42
Superior cerebellar peduncle	0.380 (0.082)	0.407 (0.076)	0.390 (0.071)	0.391 (0.076)	0.509	0.04
Thalamus	0.323 (0.021)	0.322 (0.017)	0.317 (0.022)	0.320 (0.020)	0.541	0.26
Caudate nucleus	0.200 (0.028)	0.221 (0.034)	0.219 (0.039)	0.213 (0.035)	0.082	0.06
Pallidum	0.368 (0.063)	0.401 (0.048)	0.428 (0.053)	0.400 (0.061)	<0.001	0.53
Midbrain	0.443 (0.028)	0.461 (0.026)	0.438 (0.034)	0.446 (0.031)	0.032	0.75
Pons	0.514 (0.025)	0.523 (0.018)	0.473 (0.043)	0.500 (0.039)	<0.001	1.42

Group differences were calculated using linear model analysis of variance. Effect sizes are presented as Cohen's d.

Abbreviations: HC, healthy controls; SD, standard deviation; PD, Parkinson's disease; MSA, multiple system atrophy.

### 3.3 Classification Accuracy

Based on a C5.0 decision tree algorithm generated from DTI and volume measurements, increased MD values of the MCP and the putamen were the most discriminative metrics differentiating MSA from PD. Increased apparent diffusion coefficient (ADC) values (exploiting the corresponding cut‐off values of the decision tree) in the MCP were present in all patients with cerebellar‐predominant MSA and in 68.4% of patients with Parkinson‐predominant MSA, whereas putaminal ADC increases were found in 84.2% of patients with Parkinson‐predominant MSA and 33.3% of patients with cerebellar‐predominant MSA. Cross‐validation of the classification model yields a Cohen's κ of 0.823 and an overall diagnostic accuracy of 0.914. The final decision tree is presented in the Figure S1.

## 4 Discussion

In the present study, automated analysis of the MCP and putamen revealed significant differences of DTI metrics between patients with PD and MSA with larger effect sizes compared to volumetric measures of these structures. Notably, an attribute selection by a C5.0 decision tree, which was informed by volumetric MRI as well as DTI, identified increased MD values within the putamen and the MCP as the most predictive features for a diagnosis of MSA. The overall accuracy of image classification relative to the final clinical diagnosis based on cross‐validation was 91.4%. This study extends previous research by demonstrating that automated analysis of DTI appears to be more sensitive to disease‐specific tissue changes than volumetric measures and therefore more relevant for diagnostic purposes in the early stages of the disease.

### 4.1 Diagnostic Accuracy of Microstructural Changes

Detection of structural abnormalities within subcortical brain regions yields high diagnostic accuracy for a diagnosis of MSA. Automated segmentation of structural MRI with consecutive subcortical volume calculation and adjustment for total intracranial volume further increased the diagnostic accuracy for discriminating MSA and PD.^8^, ^9^

In addition, increased MD values were shown to be a useful measure of microstructural changes in neurodegenerative disorders.^13^, ^27^, ^28^ In patients with MSA, previous DTI studies reported increased MD and reduced FA values in brain regions that are affected by MSA pathology.^20^ Interestingly, a case report of a patient with Parkinson‐predominant MSA who had serial, multimodal MRIs during the course of 5 years showed that DTI changes preceded volume loss.^29^ Furthermore, other MRI studies in patients with MSA supported this notion, suggesting that changes in diffusivity emerge prior to regional, disease‐specific brain atrophy.^27^, ^28^, ^29^, ^30^ Such findings were also reported in other neurodegenerative diseases, including Alzheimer's disease. A recent study in Alzheimer's disease mutation carriers suggested that regionally selective white matter degeneration as measured by DTI occurs years before the estimated onset of clinical symptoms.^31^ Another advantage of DTI over MRI volumetry is that DTI sequences do not require whole‐brain volume correction, which facilitates data analysis. The delineation of subcortical structures on DTI sequences, however, requires expert knowledge and extensive training of the reader. Moreover, it is time consuming and prone to variability of metric parameters because of inconsistencies regarding the delineation of boundaries and selection of adequate ROI size and shape. Such difficulties can be resolved by extending a well‐established, automated MRI segmentation pipeline with DTI to T1‐weighted image coregistration that transfers the anatomic information from high‐resolution 3‐dimensional T1 images to lower resolution DTI acquisitions.

In the present study, the MD of the MCP yielded the highest effect size in differentiating patients with PD and MSA. Accordingly, increased MCP diffusivity was selected as the most predictive measure in our diagnostic decision algorithm. This is not surprising given the results of previous research with manual ROI‐based DTI measurements and voxel‐wise group comparisons demonstrating that increased diffusivity in the MCP is common in MSA.^11^, ^17^, ^28^, ^32^, ^33^, ^34^, ^35^, ^36^ The second most discriminative feature in the present diagnostic algorithm represented putaminal MD increases. This observation was also to be expected because previous studies that manually delineated the putamen on DTI images constantly showed increased diffusivity within the putamen in patients with MSA.^17^, ^19^, ^20^, ^28^, ^34^, ^35^, ^36^, ^37^

One of the particular strengths of the present study is the observer‐independent ROI placement. Standardized, observer‐independent placement of the ROIs could be helpful in harmonizing results and might provide better test–retest reliability.

Axial and radial diffusivity measures were considered less helpful for the present study because those are 2‐dimensional measures within a single voxel and therefore inappropriate for averaged volume measurements.

### 4.2 Limitations

Some limitations need to be considered when interpreting the results of the present study. Despite the long follow‐up period and the application of operational diagnostic criteria, the lack of postmortem verification remains a potential limitation because clinical misdiagnosis cannot be fully excluded. Although automated segmentation expedites the volume measurements of brain regions, visual inspection by an expert human reader is inevitable to ensure that no misalignment of brain structures had occurred. Moreover, automated volumetry and extraction of DTI data are labor‐intensive techniques that require high‐level technology and expertise that currently limits the availability of these imaging approaches to specialized centers. Thus, future efforts are required to move the innovative diagnostic procedure to general clinical practice.

### 4.3 Conclusions

Overall, the present study confirms the excellent diagnostic accuracy of DTI measures and demonstrates that observer‐independent diffusion‐imaging analysis is feasible and reliable in the differential diagnosis of MSA versus PD. Future studies are needed to validate diagnostic algorithms in independent data sets and describe the benefits of different approaches.

## 5 Author Roles

(1) Research Project: A. Conception, B. Organization, C. Execution; (2) Statistical Analysis: A. Design, B. Execution, C. Review and Critique; (3) Manuscript: A. Writing of the First Draft, B. Review and Critique.

F.K.: 1A, 1B, 1C, 2A, 2B, 3A

V.B.: 1A, 1B, 1C, 2A, 2B, 3A

K.S.: 1A, 1B, 1C, 2C, 3B

C.M.: 1A, 1B, 1C, 2C, 3B

G.G.: 1A, 2A, 2C, 3B

E.R.G.: 1C, 2C, 3B

G.K.W.: 1C, 2C, 3B

W.P.: 1A, 1C, 2C, 3B

C.S.: 1A, 1B, 1C, 2A, 2B, 3A

## 6 Financial Disclosures of all authors (for the preceding 12 months)

F.K., V.B., C.M. and G.G. have nothing to disclose. K.S. reports grants from Oesterreichische Nationalbank, FWF Austrian Science Fund, Michael J. Fox Foundation, and International Parkinson and Movement Disorder Society and personal fees from International Parkinson and Movement Disorder Society, Boehringer Ingelheim, UCB, Lundbeck, Teva, Abbvie, and AOP Orphan Pharmaceuticals AG. G.K.W. reports receiving consulting and/or lecture fees from Affiris, Astra Zeneca, Boehringer Ingelheim, Ever Pharma, Lundbeck, Neuropore, Orion, and UCB as well as grant support from Medical University Innsbruck, Oesterreichische Nationalbank, FWF Austrian Science Fund, US MSA Coalition, Affiris, Astra Zeneca, and Boehringer Ingelheim. W.P. has received consultancy and lecture fees in relation to clinical drug programs for PD from AbbVie, AstraZeneca, BIAL, Biogen, Britannia, Grünenthal, Intec, Ipsen, Lundbeck, Novartis, Neuroderm, Orion Pharma, Prexton, Roche, Sunovion, Sun Pharma, Takeda, Teva, UCB, and Zambon and royalties from Thieme, Wiley Blackwell, Oxford University Press, and Cambridge University Press. C.S. reports research grants from the Austrian Wirtschaftservice and the Federal Ministry of Education, Science and Research of the Republic of Austria.

## Supporting information


**Figure S1** Decision tree.
