# Supplementary figures and images for: Automated Analysis of Diffusion‐Weighted Magnetic Resonance Imaging for the Differential Diagnosis of Multiple System Atrophy from Parkinson's Disease
Source: Mov Disord. 2020 Sep 16;36(1):241–5. doi: 10.1002/mds.28281 (PMC7891649; doi:10.1002/mds.28281)

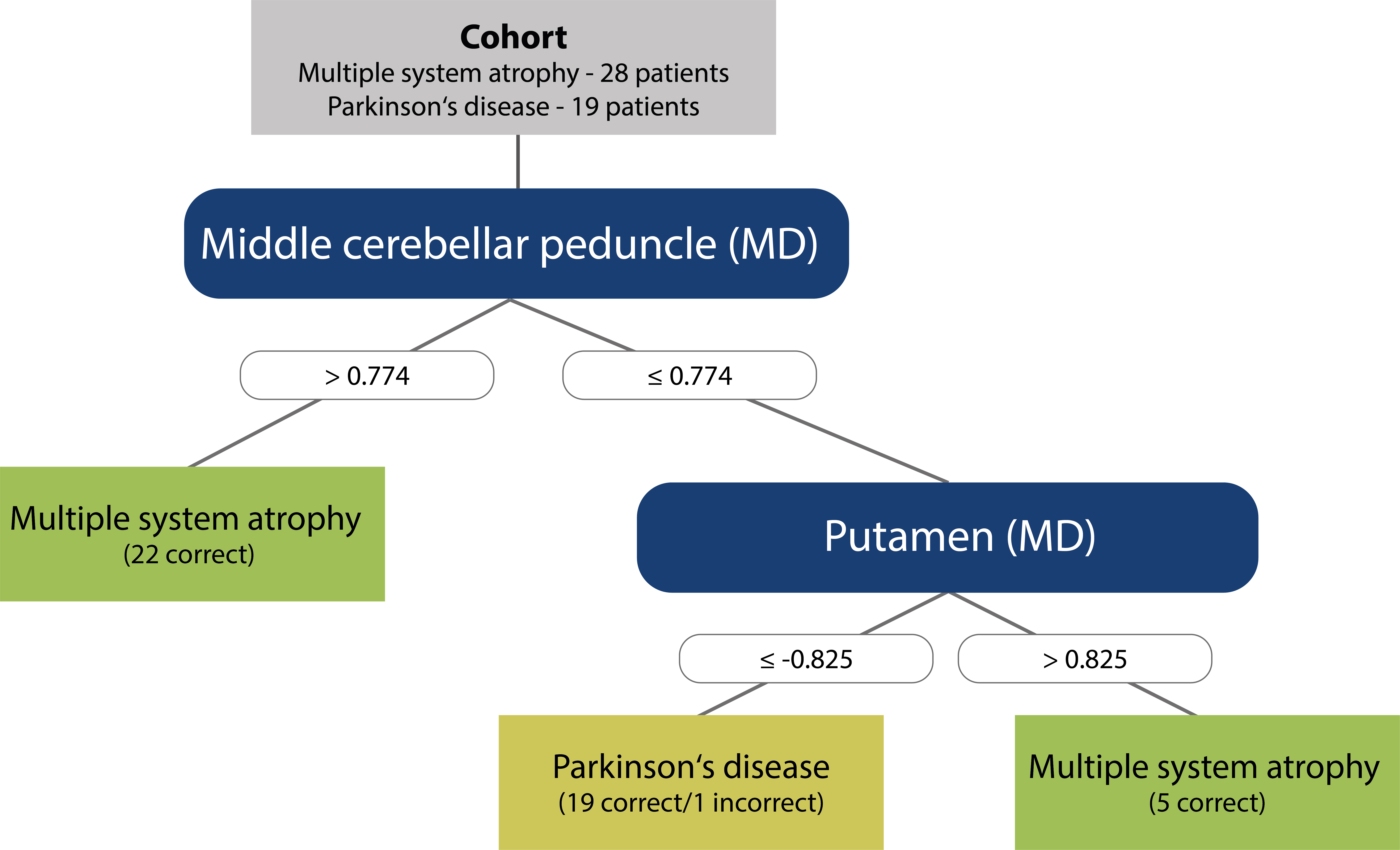

Supplement: Supplementary file 1 — Figure S1 Decision tree. [file MDS-36-241-s001.jpg]
